# Colonic Actinomycosis Presenting One Year After Partial Sigmoid Colectomy

**DOI:** 10.7759/cureus.23954

**Published:** 2022-04-08

**Authors:** Abraheim Al-nasseri, Walaa Hammad, Islam Younes, Nikhita Sachdeva, Sherif Elkattawy, Ahmed Omran, Ahmed Mowafy, Heidi Fish

**Affiliations:** 1 Internal Medicine, St. George's University School of Medicine, Elizabeth, USA; 2 Pathology and Laboratory Medicine, Ain Shams University Hospital, Cairo, EGY; 3 Internal Medicine, Trinitas Regional Medical Center/RWJBarnabas Health, Elizabeth, USA; 4 Internal Medicine, St. George's University, Union, USA; 5 Internal Medicine, Trinitas Regional Medical Center, Elizabeth, USA; 6 Clinical Research, University of Louisville, Louisville, USA; 7 Pathology and Laboratory Medicine, Trinitas Regional Medical Center/RWJBarnabas Health, Elizabeth, USA

**Keywords:** branching filamentous organisms, intrabdominal infection, sulfur granules, colon polyps, actinomycosis

## Abstract

Actinomycetes are commensal inhabitants of the oral cavity and gastrointestinal tract that can acquire pathogenicity through invasion of injured mucosa. Appendix and ileocecal regions are the most common sites involved in abdominal actinomycosis. We report a case of unusual site actinomycosis, in the recto-sigmoid colon, presenting with abdominal pain and diarrhea after one year of partial sigmoid colectomy. A colonoscopy was done, which showed a 21 mm polypoid partially obstructing mass in the recto-sigmoid colon. Histopathology showed granulation tissue with severe acute inflammation, fibrinopurulent debris with areas of abscess, and branching filamentous organisms with sulfur granules consistent with actinomycosis. Abdominal actinomycosis can infect almost all organs within the abdominal cavity; however, it is more common around the ileocecal region.

## Introduction

Actinomycetes are inhabitants of the oral cavity and gastrointestinal tract. Nearly 50% of cases of actinomycosis are caused by predisposing factors such as disruption of the mucosa, foreign bodies, and immunosuppression (i.e., chemotherapy, steroids, HIV, and diabetes). The remaining 50% of cases are reported to be idiopathic [1]. Appendix and ileocecal regions are the most common sites involved in abdominal actinomycosis. Actinomycosis commonly has an insidious presentation as the infection creates a slowly growing inflammatory mass that results in nonspecific signs and symptoms [2]. Although colonoscopy usually shows polyps or inflammatory mass in intestinal actinomycosis, pathologic identification of the actinomycosis sulfur granules or culture of the actinomycetes is the definitive diagnostic tool. Long-term antibiotics and surgical drainage are the main treatment line. We report a case of unusual site actinomycosis, in the recto-sigmoid colon, presenting with abdominal pain and diarrhea after one year of partial sigmoid colectomy.

## Case presentation

**Figure 1:**
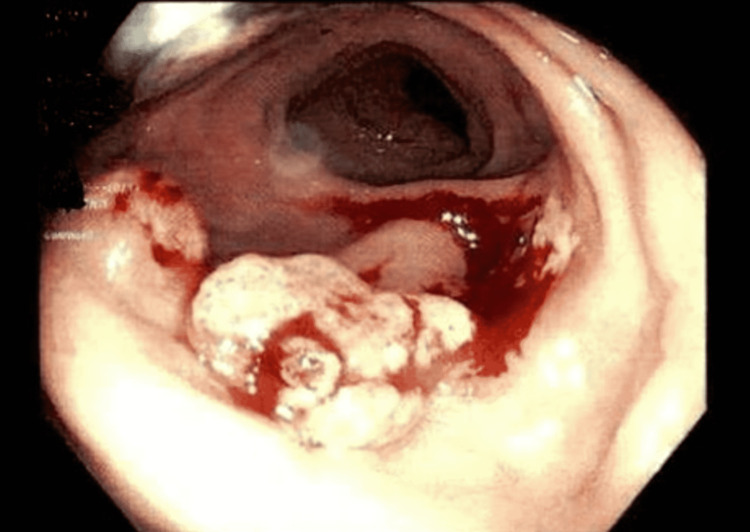
Colonoscopy showed a 20 mm polyp in the recto-sigmoid colon.

**Figure 2:**
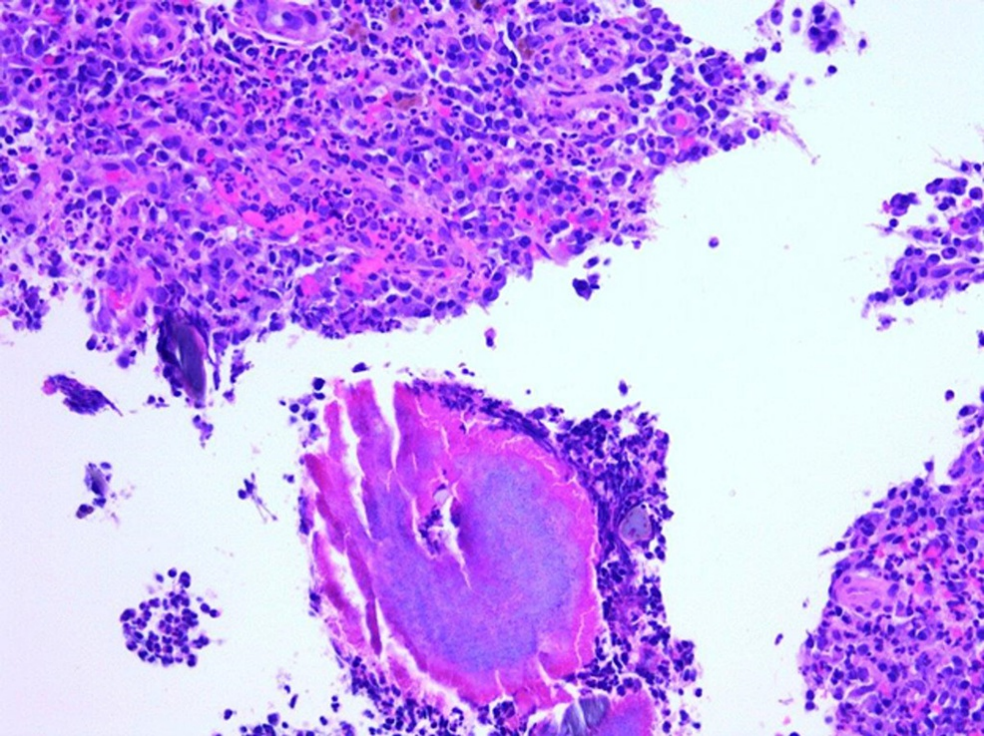
Histopathology showed granulation tissue, severe acute inflammation, fibrinopurulent debris, and branching filamentous organisms with sulfur granules consistent with actinomycosis. No dysplasia or carcinoma was present.

**Figure 3:**
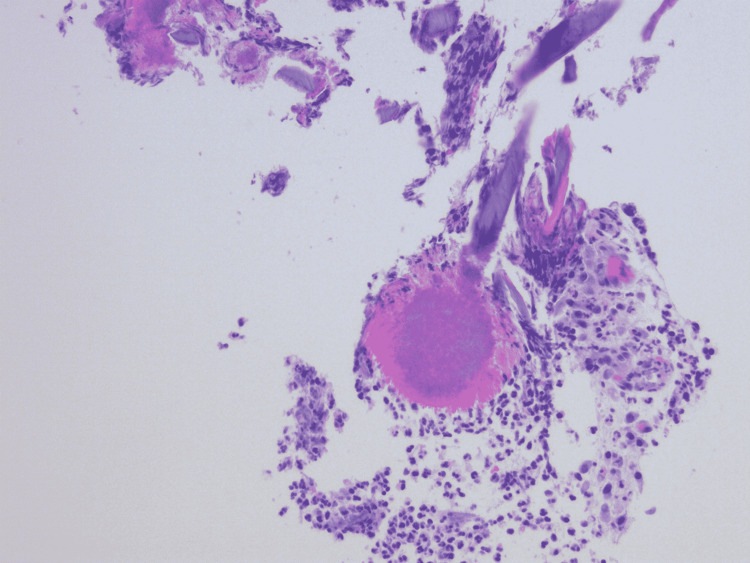
Histopathology showed sulfur granules surrounded by actinomycetes.

A 57-year-old Hispanic male with a past medical history of hypertension, diabetes mellitus, chronic kidney disease, and complicated diverticulitis status post elective partial sigmoid resection one year ago presented with a one-month history of persistent abdominal pain, nausea, and greenish diarrhea. He denied any hematochezia, melena, mucous in stool, or vomiting. On review of systems, the patient denied chest pain, shortness of breath, dizziness, fever, chills, cough, and hematuria. The patient denied any use of alcohol, smoking, or recreational illicit drugs. He denied any pertinent family history. He was afebrile (98.3°F), with a pulse of 70 beats per minute, blood pressure of 119/65 mmHg, respiratory rate of 18 breaths/minute, and O2 saturation of 97% on room air. The physical exam showed a soft non-distended abdomen and normoactive bowel sounds. However, the patient was positive for mild left lower quadrant tenderness with no rebound tenderness or rigidity. His hematological investigations were unremarkable except for creatinine level of 3.0 mg/dL, blood urea nitrogen (BUN) of 51 mg/dL (patient’s baseline), hemoglobin of 12.5 gm/dL, and mean corpuscular volume (MCV) of 91 fL. Colonoscopy showed a 21 mm polypoid partially obstructing mass in the recto-sigmoid colon distal to the patent end-to-end colo-colonic anastomosis (22 cm from the anus) (Figure 1). Histopathology showed granulation tissue with severe acute inflammation (Figure 2), fibrinopurulent debris with areas of abscess, and branching filamentous organisms with yellow color sulfur-like granules (Figure 3) consistent with actinomycosis. Immunochemical staining for cytomegalovirus (CMV) and herpes simplex virus type 1 (HSV1) was negative as well as periodic acid-Schiff (PAS) stain for other fungal organisms was also negative. No dysplasia or carcinoma was present. The patient was then treated with oral amoxicillin for six weeks.

## Discussion

*Actinomyces* is a gram-positive facultative-anaerobe bacillus that forms branching filaments and sulfur granules. Specific species can be found endogenously throughout the gastrointestinal tract, skin, and vagina. Abdominal actinomycosis is rare and consists of approximately 20% of all actinomycosis cases, whereas cervicofacial actinomycosis is the most common form of the disease, compromising about 60% of cases. Thoracic actinomycosis constitutes the remaining 15% of cases [1]. *Actinomyces israelii* is the most common human pathogen, which was found in our patient.

Disruption of the gastrointestinal mucosa from diverticulitis, surgical trauma, perforation, and colitis results in the predisposition of abdominal actinomycosis [2]. We report a case of recto-sigmoid colon actinomycosis in a 56-year-old diabetic male with a history of complicated diverticulitis status post elective partial sigmoid resection one year ago. *Actinomyces* is an endogenous microorganism of the digestive system and requires a significant compromise of the normal mucosa to cause an infection. Nearly 50% of cases of actinomycosis are caused by predisposing factors such as surgical trauma, appendicitis, diverticulitis, perforation, neoplasia, foreign bodies, and immunosuppression (i.e., chemotherapy, steroids, HIV, and diabetes). The remaining 50% of cases are reported to be idiopathic [2]. The pathophysiology of infection by *Actinomyces* remains unclear [3]. Abdominal actinomycosis can infect almost all organs within the abdominal cavity; however, it remains unclear why some organs are more susceptible than others. Additionally, the clinical presentation differs with respect to the abdominal organ involved. Actinomycosis more commonly affects the right side of the colon, especially around the ileocecal region [4]. Abdominal actinomycosis can involve the liver and produce multiple hepatic abscesses. Hepatic actinomycosis accounts for 5%-15% of cases and has often been noted to arise after an initial abdominal infection from a different organism [2].

An insidious presentation is common as the infection from *Actinomyces* creates a slowly growing inflammatory mass that results in nonspecific signs and symptoms. Similar to our patient, the initial presentation typically manifests itself with abdominal pain, nausea, and altered bowel habits. These nonspecific findings make differential diagnoses of abdominal actinomycosis more challenging, as more common diagnoses must be ruled-out (i.e., colitis, inflammatory bowel disease, colonic malignancy, diverticulitis, appendicitis, irritable bowel disease, and pelvic inflammatory disease) [5].

Colonoscopy is useful in ruling-out malignancy or other inflammatory pathologies in these cases and confirming the diagnosis with histological testing of the collected specimens [6]. Since actinomycosis is known to originate outside of the mucosa, diagnostics imaging such as a CT scan can be useful in visualizing other organs involved. The most effective method in diagnosing actinomycosis has been reported with the use of CT-guided drainage of abscesses followed by histological evaluation [7]. The typical histologic finding is the sulfur granules (containing actinomycetes and calcium phosphate), which can be viewed using Grocott-Gomori staining [8].

Antibiotics have shown to be effective in uncomplicated cases. Penicillin or cephalosporin is taken at high doses for at least six months or until the effective dissolution of the lesion remains the standard treatment. Clinically significant treatments have also been reported using tetracycline, erythromycin, chloramphenicol, clindamycin, and imipenem and become especially useful in patients with penicillin allergy. When combined with surgical resection, a cure rate of approximately 90% can be achieved [9].

## Conclusions

Abdominal actinomycosis is not a common intra-abdominal infection and requires a high level of suspicion, especially in high-risk patients such as those with a history of previous abdominal surgery. Long-term antibiotic treatment is usually an effective treatment for such cases, and surgical drainage may be required in complicated cases.

## References

[1] Kaya E, Yilmazlar T, Emiroğlu Z, Zorluoğlu A, Bayer A (1995). Colonic actinomycosis: report of a case and review of the literature. Surg Today.

[2] Privitera A, Milkhu CS, Datta V, Rodriguez-Justo M, Windsor A, Cohen CR (2009). Actinomycosis of the sigmoid colon: a case report. World J Gastrointest Surg.

[3] Zamani F, Sohrabi M (2015). Clinical, endoscopic, and histopathological aspects of sigmoid actinomycosis; a case report and literature review. Middle East J Dig Dis.

[4] Thanos L, Mylona S, Kalioras V, Pomoni M, Batakis N (2004). Ileocecal actinomycosis: a case report. Abdom Imaging.

[5] Yang SS, Im YC (2018). Severe abdominopelvic actinomycosis with colon perforation and hepatic involvement mimicking advanced sigmoid colon cancer with hepatic metastasis: a case study. BMC Surg.

[6] Valour F, Sénéchal A, Dupieux C (2014). Actinomycosis: etiology, clinical features, diagnosis, treatment, and management. Infect Drug Resist.

[7] Kim JC, Ahn BY, Kim HC, Yu CS, Kang GH, Ha HK, Lee MG (2000). Efficiency of combined colonoscopy and computed tomography for diagnosis of colonic actinomycosis: a retrospective evaluation of eight consecutive patients. Int J Colorectal Dis.

[8] Smith TR (1992). Actinomycosis of the distal colon and rectum. Gastrointest Radiol.

[9] García-García A, Ramírez-Durán N, Sandoval-Trujillo H, Romero-Figueroa MDS (2017). Pelvic actinomycosis. Can J Infect Dis Med Microbiol.

